# Prevalence of Transcriptionally Active HPV Infection in Tumor-Free Oropharyngeal Tissue of OPSCC-Patients

**DOI:** 10.3389/fonc.2022.835814

**Published:** 2022-04-22

**Authors:** Vittoria Guarda, Lea Schroeder, Michael Pawlita, Kristian Ikenberg, Niels J. Rupp, Wolfram Jochum, Sandro J. Stoeckli, Dana Holzinger, Martina A. Broglie

**Affiliations:** ^1^ Department of Otorhinolaryngology, Head and Neck Surgery, University Hospital Zurich, Zurich, Switzerland; ^2^ Infections and Cancer Epidemiology, Infection Inflammation and Cancer Program, German Cancer Research Center (DKFZ), Heidelberg, Germany; ^3^ Department of Pathology and Molecular Pathology, University Hospital and University of Zurich, Zurich, Switzerland; ^4^ Institute of Pathology, Kantonsspital St. Gallen, St. Gallen, Switzerland; ^5^ Department of Otorhinolaryngology, Head and Neck Surgery, Kantonsspital St. Gallen, St. Gallen, Switzerland

**Keywords:** head and neck cancer, oropharynx cancer, human papillomavirus, second primary neoplasms, human papillomavirus DNA tests

## Abstract

**Objectives:**

The natural history of HPV-related oropharyngeal squamous cell carcinoma (OPSCC) is still largely unknown. Since reports of second primary tumors (SPTs) in patients with HPV-related OPSCCs are increasing, a multifocal HPV infection, hinting a «virus-induced field effect», has been hypothesized. This study aimed to investigate the HPV-prevalence in normal appearing oropharyngeal tissue in patients with OPSCCs.

**Materials and Methods:**

49 OPSCC patients undergoing panendoscopy were prospectively enrolled. Tumor specimens and biopsies of normal appearing oropharyngeal tissue adjacent to and distant from the index OPSCC underwent histopathological examination, p16^INK4A^ immunohistochemical staining, HPV DNA and mRNA-detection. Patient characteristics and follow-up data on SPTs were obtained.

**Results:**

26 of 49 (53%) OPSCC were positive for HPV DNA and p16^INK4A^. HPV mRNA was detected in 23 of 26 (88%) of these tumor samples. HPV DNA was detected in 36% adjacent mucosa and in 17% distant mucosa samples and only in patients with an HPV-related index OPSCC. HPV mRNA could not be detected in tumor-free distant and adjacent mucosa samples. No evidence of association between HPV detection in normal appearing mucosa and development of second primary tumors was found.

**Conclusions:**

HPV was detectable but not transcriptionally active in adjacent/distant tumor-free oropharyngeal tissue. This suggests that a multifocal HPV infection, hinting a «virus-induced fielcd cancerization», may not be pertaining to HPV-related OPSCC.

## 1 Introduction

A transcriptionally active infection with high risk human papillomavirus (HR-HPV) is now established as a major risk factor for the development of oropharyngeal squamous cell carcinomas (OPSCCs) (1, 2). HPV-related OPSCCs are associated with a better treatment response and improved outcome compared to their tobacco- and alcohol-induced counterparts (3, 4). Moreover, compelling evidence demonstrates a lower risk of developing second primary tumors (SPT) in these patients, possibly contributing to their superior overall and disease-free survival (5–8). SPTs represent, in fact, one of the leading causes of death in head and neck cancer patients and typically arise within the upper aerodigestive tract (UADT) (9). The development of SPTs has been linked to the concept of «field cancerization», which describes multifocal alterations of mucosa “fields” following a progressive, alcohol- and tobacco-induced accumulation of adverse genetic modifications (10). Nevertheless, some cases of synchronous and metachronous HPV-driven SPTs have been recently described (11–17), raising the question of a possible, multifocally persistent oncogenic infection with HR-HPV in the oropharyngeal mucosa.

The mechanism underlying the transformation from a transient oropharyngeal HPV infection to virus-induced cancerization is still largely unknown (18). In fact, while HPV DNA has been frequently detected in dysplastic tonsillar epithelium (19), several studies reported a moderate (6.9% - 13.1%) to low (0 - 6.3%) HR-HPV prevalence in respective oral gargles (20, 21) and non-malignant tonsillar tissue (22–26). Thus, detection of transcriptionally active HR-HPV in normal appearing mucosa may indicate areas of «virus-induced field cancerization».

The aim of this study was to prospectively and systematically assess the prevalence of a transcriptionally active HR-HPV infection in the normal appearing mucosa adjacent to and distant from the tumor in patients with OPSCCs and its potential impact on development of SPTs.

## 2 Materials and Methods

### 2.1 Patients and Sample Collection

Details on the patients’ cohort have been previously published (27). Briefly, patients with oropharyngeal squamous cell carcinoma (OPSCC) undergoing panendoscopy were prospectively enrolled at Kantonsspital St. Gallen. The study was approved by the local ethics committees (EKSG 09/124’) and written informed consent was obtained from all patients prior to study entry. Patient characteristics and follow-up data were obtained from questionnaires and clinical charts. Follow-up time (months) was reported after completion of therapy. In the present analysis, second primary tumors (SPT) from the upper aerodigestive tract (UADT) comprising the oral cavity, oropharynx, hypopharynx, larynx and esophagus were considered. Tumors that have been diagnosed more than six months apart from time of diagnosis of the index tumor were defined as metachronous (11).

During panendoscopy, iodine solution for mucosal disinfection was used. Biopsies (approximately 3 mm) were taken from the tumor, from the normal appearing mucosa adjacent to the tumor and from macroscopically normal appearing contralateral tonsil tissue at least 10 mm distant from the tumor. In order to avoid cross-contamination sampling was performed in the following sequence: contralateral – adjacent – tumor tissue and instruments were replaced after each biopsy. Tumor biopsies were primarily used for diagnostic purposes; fractions of these specimens were preserved for further molecular analysis in the context of this study. HPV tumor status by HPV DNA and p16^INK4A^ immunohistochemical staining (IHC) was assessed in diagnostic biopsies and in surgical specimens of the tumor, if resection was performed.

### 2.2 Histopathological Examination and p16^INK4A^ Immunohistochemical Staining

Tissue specimens were available as formalin-fixed paraffin-embedded (FFPE) blocks. FFPE tissue sections were prepared using a microtome. The first and the last 4 µm section from each biopsy were prepared for hematoxylin/eosin (HE) staining. In between, 3 x 10 µm curls were sectioned for DNA and RNA isolation, respectively, and one 4 µm section was prepared for p16^INK4A^ immunohistochemical staining. During sectioning of FFPE tissues, an established cleaning protocol was applied in order to prevent cross-contamination between samples. The microtome was extensively cleaned with acetone, ethanol and RNase AWAY^®^ (Molecular Bio-Products, Inc., San Diego, CA, USA) before and after each biopsy and blades and gloves were replaced. After five to ten patient tissues, a mouse brain tissue was processed to monitor potential cross-contamination.

Histopathological analysis for presence of tumor cells or dysplasia was independently performed by two pathologists (KI, NJR) on hematoxylin/eosin stained sections. p16^INK4A^ immunohistochemical staining was performed on an automated staining system (Ventana BenchMark ULTRA, Roche-Ventana Medical System, Tucson, AZ, USA) according to the manufacturer’s instructions. After pretreatment with Cell Conditioner Solution (CC1) for 48 min, mouse monoclonal antibody (anti-p16^INK4A^, clone E6H4, ready to use) was applied for 4 min (Roche Diagnostics, Rotkreuz, Switzerland). p16^INK4A^-positivity, was defined as unequivocal nuclear and cytoplasmic staining in at least 70% of the tumor cells (28).

### 2.3 Isolation of Nucleic Acids

Genomic DNA was isolated from tissue sections by incubating for 16 h at 56°C in 200 µl of proteinase K solution (1 mg/ml, 45mM Tris-HC, 0.9mM EDTA, 0.45% Tween 20). This incubation was followed by enzyme inactivation for 10 min at 72°C and centrifugation. The aqueous phase containing the DNA was transferred into a nuclease-free tube. To reverse potential cross-linking, the isolated DNA was incubated for 20 min at 90°C, centrifuged and transferred into a new tube. For PCR, 5 µl of DNA was used. Total RNA was isolated using the Pure-Link FFPE Total RNA Isolation Kit (Invitrogen, Thermo Fisher Scientific, Waltham, Massachusetts, USA) with an additional DNase treatment for 15 minutes at room temperature prior to elution in 50 µl RNase-free water. The PCR was performed with 1 µl of RNA.

### 2.4 HPV DNA and RNA Assays

HPV DNA was detected in biopsies using a previously described multiplex HPV genotyping (MPG) assay that includes a broad-spectrum general primers (BSGP5+/6+)-PCR amplifying the L1 region (~150 bp) of 51 mucosal HPV types, as previously described by Schmitt et al. (29). PCR products were detected by hybridization to HPV type-specific probes coupled to fluorescently labelled xMAP Luminex beads (29, 30). Human beta-globin served as internal control and samples that were negative for both HPV and beta-globin were excluded from the analysis (invalid). The E6*I transcripts of the HPV types 16 and 33 (identified in the MPG assay) were assessed by reverse-transcriptase PCR generating short amplicons (65 and 74 bp, respectively) and hybridization as previously described (31). RNA integrity was assessed by co-amplification of ubiquitin C mRNA (85 bp). Samples positive for HPV and/or ubiquitin C were valid.

### 2.5 Statistical Analysis

Fisher’s exact test was applied for testing relationships between categorical variables using the GraphPad Prism 8 software. P values <0.05 were considered statistically significant.

## 3 Results

### 3.1 Patients’ Demographics

Demographic data are displayed in **Table 1**. The mean age at diagnosis was 61.6 years. There were more male than female patients in our population. Eight (16%) patients had surgery only. Surgery followed by adjuvant radiotherapy/radiochemotherapy was performed in 24 (49%) cases, whereas 17 (35%) patients underwent primary radiotherapy/radiochemotherapy. Follow-up data and data on SPTs are presented in paragraph 3.4. The HPV status of the SPTs was not available for analysis.

**Table 1:** Selected demographic data and characteristics of study participants.

		No.	%
**Total patients**		49	100
**Age (years)**	Range	29-81	
	Median	62	
**Gender**	Male	38	78
	Female	11	22
**Tobacco Smoking (>10 pack years)**	Yes	30	61
	No	16	33
	Unknown	3	6
**Alcohol use (>3 Units)**	Yes	19	39
	No	30	61
**Lifetime sexual partners**	0-9	23	47
	10-19	11	22
	≥ 20	11	22
	Unknown	4	8
**Tumor site**	Tonsil	30	61
	Base of tongue	12	24
	Other subsites	7	14
**Stage**	Stage I/II (p16^INK4A^-negative)	5	10
	Stage I/II (p16^INK4A^-positive)	24	49
	Stage III/IV (p16^INK4A^-negative)	14	29
	Stage III/IV (p16^INK4A^-positive)	6	12
**T stage**	T1-T2 (p16^INK4A^-negative)	10	20
	T1-T2 (p16^INK4A^-positive)	22	45
	T3-T4 (p16^INK4A^-negative)	9	18
	T3-T4 (p16^INK4A^-positive)	8	16
**N stage**	Nx/N0 (p16^INK4A^-negative)	9	18
	Nx/N0 (p16^INK4A^-positive)	5	10
	N1-N3 (p16^INK4A^-negative)	10	20
	N1-N3 (p16^INK4A^-positive)	25	51
**Follow-up time (months)**	Range	0-101	
	Median	58	

### 3.2 HPV Detection in Tumor Tissue

HPV-positive OPSCC were defined by double positivity for both p16^INK4A^ immunhistochemical staining and HPV DNA by BSGP5+/6+-PCR. In brief, 26 (53%) tumors were attributable to HPV, comprising 22 (85%) tumors driven by HPV16 and four (15%) tumors driven by HPV33. All other tumors were defined as non-HPV-related (**Table 2**).

**Table 2:** HPV status in tumor tissue.

	No. of cases (%)
**HPV-related OPSCCs**	**26 (53%)**
HPV16 DNA pos. + p16 ^INK4A^ IHC pos.	22 (45%)
HPV33 DNA pos. + p16 ^INK4A^ IHC pos.	4 (8%)
	
**HPV-negative OPSCCs**	**23 (47%)**
HPV16 DNA pos. + p16 ^INK4A^ IHC neg.	3 (6%)
HPV DNA neg. + p16 ^INK4A^ IHC pos.	4 (8%)
HPV DNA neg. + p16 ^INK4A^ IHC neg.	16 (33%)

OPSCCs, oropharyngeal squamous cell carcinomas; IHC, immunohistochemistry.

**Figure 1:**
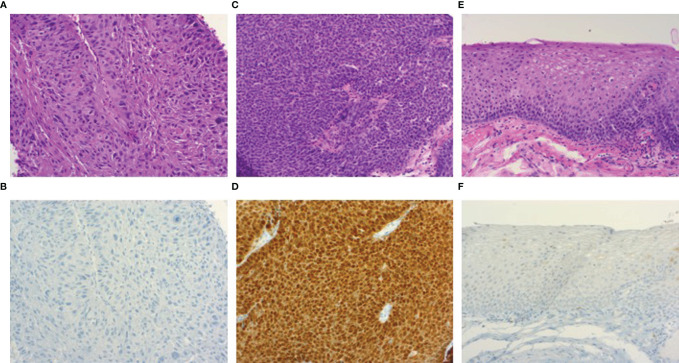
Photomicrographs of hematoxylin/eosin and p16^INK4A^ immunohistochemical stained sections (100x). HPV-negative oropharyngeal squamous cell carcinoma **(A)** showing p16^INK4A^ negativity by immunohistochemistry **(B)**. Image **(C)** demonstrates an HPV-related oropharyngeal squamous cell carcinoma showing p16^INK4A^ overexpression **(D)**, accompanied by a section of dysplasia-free adjacent mucosa **(E)** displaying negativity for p16^INK4A^ **(F)**.

Forty-nine (100%) samples detached from diagnostic tumor biopsies were available for HPV mRNA analysis. HPV16 or HPV33 mRNA was detected in 23 of 26 (88%) samples of tumors presenting both HPV DNA positivity and p16^INK4A^ overexpression in the diagnostic biopsies or surgical specimens (**Figure 1**). The remaining three (12%) HPV mRNA-negative study samples showed no dysplasia as well as p16^INK4A^ negativity in the histological examination. Finally, no HPV mRNA was found in tumors defined as non-HPV-related.

### 3.3 Histological Examination, p16^INK4A^ Immunohistochemistry and HPV Detection in Normal Appearing Mucosa Adjacent to and Distant From the Tumor

Forty-eight samples from normal appearing mucosa close to the tumor and 45 ones from normal appearing oropharyngeal tissue distant from the tumor were available for histological examination, p16^INK4A^ immunohistochemistry, HPV DNA as well as HPV mRNA analysis. Matching results are displayed in **Table 3**. Ten samples (5 adjacent ones, 5 distant ones), labeled as invalid, failed the multiplex HPV genotyping assay, since they tested negative for both HPV and human beta-globin. No invalid result was observed for HPV mRNA analysis.

**Table 3:** HPV detection in normal appearing oropharyngeal tissue adjacent to and distant from the tumor in HPV-positive and negative OPSCCs.

No.	Mucosa adjacent to the tumor				Distant mucosa			
	HPV DNA by BSGP5+/6+ PCR				HPV DNA by BSGP5+/6+ PCR			
HPV mRNA	Positive	Negative	Invalid	Total	Positive	Negative	Invalid	Total
Positive	1	0	0	1	0	0	0	0
Negative	8	34	5	47	4	36	5	45
Total	9	34	5	48	4	36	5	45

BSGP5+/6+ PCR = broad-spectrum general primers 5+/6+-PCR.

#### 3.3.1 Patients With a Non-HPV-Related Index Tumor

Among the 23 patients with a non-HPV-related index tumor, 19 (83%) samples of normal appearing mucosa adjacent to the tumor showed neither carcinoma nor high-grade dysplasia in the histological examination. Three (13%) adjacent mucosa samples presented invasive carcinoma, most likely manifestations of the primary tumors. One (4%) adjacent mucosa specimen showed high-grade dysplasia. Here, no p16^INK4A^ overexpression was observed, although p16^INK4A^ positivity was described in the index tumor.

In the distant mucosa samples’ analysis, carcinoma was detected in only one specimen, later identified as a synchronous second primary tumor of the contralateral tonsil in a patient with a Stage IV, non-HPV-related index tonsillar carcinoma. The remaining distant tissue specimens showed neither carcinoma nor high-grade dysplasia in the histological examination.

None of the adjacent and distant mucosa specimens presented p16^INK4A^ overexpression.

Neither HPV DNA nor mRNA was detected in the adjacent and distant tissue samples of the 23 patients with non-HPV-related index tumors.

#### 3.3.2 Patients With an HPV-Related Index Tumor

Among the 26 patients with an HPV-related index tumor, 25 adjacent mucosa samples and 24 distant ones were available for analysis. Twenty-four (96%) adjacent mucosa samples showed no dysplasia (**Figure 1**); in one (4%) adjacent mucosa specimen carcinoma was detected, also manifesting p16^INK4A^ overexpression. p16^INK4A^ overexpression was not found in dysplasia-free adjacent samples.

Neither dysplasia nor p16^INK4A^ overexpression were identified in distant tissue specimens.

**Figure 2:**
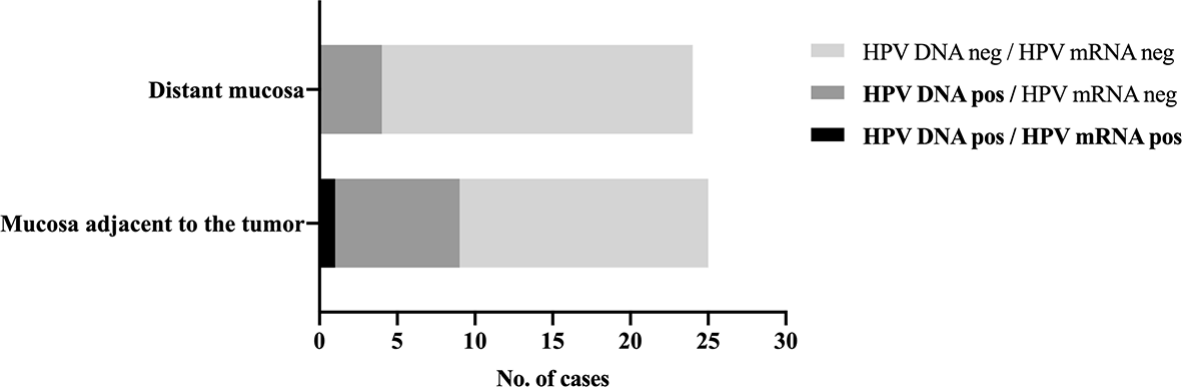
HPV detection in normal appearing mucosa adjacent to and distant from the tumor. Distribution of HPV DNA-positive and HPV mRNA-positive samples in normal appearing tissue adjacent to and distant from the tumor in patients with an HPV-related index oropharyngeal squamous cell carcinoma. HPV16 mRNA was detected in a single p16^INK4A^-positive and HPV DNA-positive adjacent mucosa sample, which also showed carcinoma in the histological examination, likely from the index tumor, suggesting sampling error.

HPV DNA was detected in nine of the 25 (36%) adjacent mucosa samples and in four (17%, 1 HPV DNA-invalid excluded) distant ones (**Figure 2**). HPV genotyping revealed the same HPV type that was present in the index tumor, which was HPV16 in all cases.

HPV16 mRNA was detected in a single (4%), HPV DNA-positive adjacent mucosa sample, which also presented carcinoma and p16^INK4A^ overexpression in the histological examination. Otherwise, all remaining adjacent and distant tissue samples tested negative for HPV16 and HPV33 mRNA.

### 3.4 Second Primary Tumors and HPV Detection

The median follow-up time of the patient cohort was 58 months (range 0-101 months). Eight (16%) patients developed at least one second primary tumor (SPT) of the upper aerodigestive tract (UADT). The median interval between diagnosis of the index tumor and onset of SPTs was 12 months (range 0-103 months). As previously stated, the HPV status of the secondary primary tumors, particularly data on p16^INK4A^ immunohistochemistry and HPV genotyping, was not available for analysis. Details on those patients are demonstrated in **Table 4**. Second primary tumors were significantly more common in patients with non-HPV-related index tumors (7/23, 30%) compared to those with HPV-related index tumors (1/26, 4%, p= 0.0210, Fisher’s exact test).

**Table 4:** Selected demographic data and characteristics of patients with a second primary tumors.

Sex/age (years)	Follow-up (months)	Index tumor HPV-status	Second primary tumor site	Onset period	Interval months	Tobacco smoking (>10 pack years)
M/54	81	HPV-negative	oral cavity	MC	12	yes
			oral cavity	MC	36	
			oropharynx	MC	73	
M/51	101	HPV-positive^a^	oropharynx	MC	90	yes
			hypopharynx	MC	103	
M/70	95	HPV-negative	oral cavity	MC	7	yes
M/61	41	HPV-negative	esophagus	S	0	yes
			oral cavity	MC	24	
M/56	8	HPV-negative	larynx	S	0	yes
M/45	20	HPV-negative	oropharynx	S	0	yes
M/49	31	HPV-negative	oral cavity	S	0	yes
F/68	51	HPV-negative	hypopharynx	S	0	yes
			oral cavity	MC	22	

F, female; M, male; MC, metachronous; S, synchronous. ^a^, as defined by double positivity for both p16^INK4A^ immunhistochemical staining and HPV DNA.

#### 3.4.1 Patients With a Non-HPV-Related Index Tumor

Among patients with non-HPV-related index tumors, five (22%) patients developed at least one SPT of the oral cavity. One (4%) patient was diagnosed with a SPT of the hypopharynx, one (4%) with a SPT of the larynx and another one (4%) with a SPT of the esophagus. Two (9%) patients had a SPT of the oropharynx: one patient was diagnosed with an index non-HPV-related carcinoma of the right soft palate and developed a carcinoma of the right base of tongue after 73 months. As previously mentioned, a synchronous second primary tumor of the contralateral tonsil was detected in a patient with an index non-HPV-related tonsillar carcinoma of the left tonsil.

#### 3.4.2 Patients With an HPV-Related Index Tumor

Among patients with an HPV-related index OPSCC, only one (4%) patient, who was initially diagnosed with an HPV-related index OPSCC of the right tonsil, developed a metachronous carcinoma of the ipsilateral base of tongue. However, no HPV DNA nor HPV mRNA were detected in the adjacent and distant mucosa samples of this patient. In this small cohort of patients with HPV-related index tumors, we did not find any evidence of link between HPV detection in normal appearing oropharyngeal tissue and development of second primary tumors.

## 4 Discussion

In the last decades, HPV-related OPSCCs have gained clinical significance, mostly due to their rising incidence and better prognosis compared to HPV-negative counterparts (3, 32). Although the understanding of the clinical and demographical profile of this disease has considerably improved over the years (33), its natural history still remains largely unknown (18). SPTs have been increasingly reported in patients with HPV-related OPSCCs, challenging the assumption of a lower risk of SPTs due to the lack of a “tobacco- and alcohol induced field cancerization” (17). It may therefore be hypothesized that detection of high-risk HPV in the oropharyngeal mucosa could indicate areas of «virus-induced field cancerization» and play a role in HPV-related SPT-carcinogenesis. In our study, we prospectively examined the prevalence of a transcriptionally active HR-HPV infection in the normal appearing oropharyngeal tissue in patients with OPSCCs and its potential impact on developing SPTs.

In our cohort, 26 out of 49 (53%) tumors have tested positive for HPV DNA plus p16^INK4A^ positive immunohistochemistry and were defined as HPV-related OPSCCs. This percentage matched current data from Germany (34) as well as data presented by Castellsagué et al. who estimated the HPV-attributable fraction for OPSCCs to be 44.9%-50% in Central-Eastern Europe (35).

Furthermore, we performed testing for HPV E6*I mRNA, since HPV mRNA detection is generally accepted as the gold standard for diagnosis of a transcriptionally active HPV infection (33, 36, 37). High HPV mRNA-detection rates (88%) in samples taken from HPV-related OPSCCs support the reliability of the algorithm comprised of p16^INK4A^ immunohistochemistry plus HPV DNA as surrogate for a transcriptionally active HPV infection in OPSCCs, which was found to have a sensitivity and specificity, respectively, of 96% and 98% (38, 39). In our cohort, however, three samples extracted from HPV-related tumors showed HPV mRNA-negativity as well as lack of high-grade dysplasia or carcinoma in the histological examination and were therefore not representative for OPSCC tissue, suggesting sampling error.

In the mucosa adjacent to the tumor, carcinoma was detected in four specimens, most likely manifestations of the primary tumors. The matching p16^INK4A^ status supports this assumption. One further adjacent mucosa specimen showed high grade dysplasia. Here, no p16^INK4A^ overexpression was detected, while p16^INK4A^ immunopositivity was described in the HPV DNA-negative index tumor, possibly indicating different neighboring dysplastic foci or intratumor heterogeneity, since this patient had history of tobacco and alcohol consumption (18, 40). Moreover, there was no p16^INK4A^ overexpression in the dysplasia-free samples adjacent to and distant from the tumor. Implications of p16^INK4A^ immunohistochemical testing in normal oropharyngeal tissue should be further inquired, since p16^INK4A^ overexpression was already detected in tumor-free tonsil samples (28%) from patients treated for chronic tonsillitis by tonsillectomy (41). Similarly, Begum et al. reported up-regulated p16^INK4A^ in nonneoplastic oropharyngeal epithelium, while HPV DNA was only found in carcinoma and dysplastic epithelium (19), questioning the suitability of p16^INK4A^ as a single marker for definition of an HPV-related OPSCC.

The HPV detection methods used in our study stood out due to their high analytical sensitivity for HPV DNA (100 and 10 plasmid copies of HPV16 and HPV33 detectable, respectively) and E6*I mRNA (10 *in vitro* transcript copies of HPV16 and HPV33 detectable) (29, 31). 10 samples failed the multiplex HPV genotyping assay, most probably reflecting poor DNA quality. No invalid result was observed for HPV mRNA analysis, since this assay was optimized for technically challenging material like archived FFPE samples by the use of ultra-short amplicons.

HR-HPV-presence as by HPV DNA detection was found in normal appearing mucosa samples (36% adjacent and 17% distant samples) of patients with an HPV-related index OPSCC, inserting in a broad landscape of data on the prevalence of oral and oropharyngeal HR-HPV infection.

According to Joseph et al., who demonstrated consensus genetic sequences of HPV16 in synchronous tonsillar carcinomas, it could be hypothesized that the same HPV infection found in tumor tissue also related to the one detected in adjacent and distant mucosal specimens, since genotyping consistently revealed HPV16 (11). Lack of HPV detection in the adjacent and distant mucosa of patients with a non-HPV-related index OPSCC also corroborates this interpretation. However, an incidental second infection with HPV16 should also be taken into consideration. In fact, several studies described a moderate (6.9% - 13.1%) to low (0 – 6.3%) HR-HPV prevalence in respective oral gargles (20, 21, 42) and non-malignant tonsillar tissue (22–26). In line with our results, a further study showed HPV DNA detection in 21% of 108 pharyngeal endoscopic biopsies of patients with an index tonsillar carcinoma (43). Nevertheless, reports on HPV prevalence are afflicted by lack of standardized detection techniques and by small sample sizes, making data interpretation very challenging. Moreover, HPV DNA analysis is usually employed as primary HPV detection method in most studies (20–26), even if HPV DNA detection may reveal a transient, rather than a transforming HPV infection (44). A recent systematic review investigated the prevalence of oral HR-HPV infection in 28544 healthy individuals and its potential influence on HPV-attributable fractions (Afs) of OPSCCs and HPV-related OPSCC rates (45). In fact, the authors could not find a significant correlation between the oral HPV prevalence and differences in HPV-Afs or HPV-related OPSCC rates across healthy populations (45).

In order to investigate the HPV transcriptional activity in the normal appearing oropharyngeal mucosa we tested for presence of HPV mRNA. Here, a single adjacent mucosa sample showed HPV16 mRNA-positivity. However, here carcinoma cells, likely from the HPV16-positive index tumor, were revealed in the histological examination, suggesting sampling error because of a larger microscopic tumor extension. Furthermore, no HPV mRNA was detected in distant tissue samples. Summarily, no evidence was found for a multifocal transcriptionally active HPV infection in tumor-free oropharyngeal tissue adjacent to and/or distant from the index tumor, corroborating and broadening previous data by Rietbergen et al., who could not find any HPV16 mRNA in tumor-free resection margins of HPV-driven OPSCCs treated by surgery (46).

In this context, the emergent number of reports about second primary tumors (SPT) in patients with HPV-related OPSCC becomes increasingly relevant, since a link between a potential multifocal HPV infection and SPTs-development has been hypothesized (11–17, 47). Indeed, as cited above, Joseph et al. detected the same HPV16 variant by E6 DNA sequencing in four pairs of HPV tonsil carcinomas, suggesting HPV multifocality (11). This evidence is challenging previous studies as well as our observations, which indicated a lower risk of development of SPT in patients with HPV-related tumors in contrast to HPV-negative OPSCCs patients, especially in never smokers (5, 7). Now, in the extensive systematic review presented by Strober et al. the prevalence of SPTs in HPV-related OPSCC patients ranged from 0.95% to 10% and the contralateral tonsil was the most common site for development of a HPV-mediated SPTs (17). Whereby, we wondered if HPV detection in tumor-free neighboring tissue and in the contralateral tonsil might correlate with the development of second primary tumors (SPT) and indicate a possible «virus-induced field effect». In the median follow-up time of 58 months, only one patient who was initially diagnosed with an HPV-related index OPSCC and had persistent smoking habits, developed SPTs during follow-up. Neither HPV mRNA nor HPV DNA could be detected in the adjacent or distant tissue biopsies of this patient. In this small cohort, we found no evidence of link between HPV detection in normal appearing mucosa and origin of second primary tumors. Unfortunately, no information about the HPV status of the SPTs (p16^INK4A^ immunohistochemistry and HPV genotyping) was available for analysis, which represents a clear limitation of this study. Moreover, the interpretation of these data is limited by a small number of participants and limited follow-up documentation. Further, only FFPE tissue samples were available for DNA and mRNA isolation, which are naturally inferior in quality compared to fresh frozen material. Since HPV-related OPSCCs are usually small and difficult to detect (48), challenging samples’ acquirement and therefore possible sampling error should also be taken into consideration. Larger cohort studies including fresh frozen material are clearly needed in order to validate this data.

In conclusion, we present a prospective cohort study addressing the prevalence of HR-HPV infection in the normal appearing oropharyngeal tissue of OPSCC-patients with a robust HPV detection methodology. We showed that HPV was detectable but not transcriptionally active in adjacent/distant tumor-free oropharyngeal tissue. In this small cohort, we found no association between HPV detection and development of SPTs. These data suggest that a multifocal transcriptionally active HPV infection, hinting a «virus-induced field cancerization», may not be pertaining to HPV-related OPSCC. Future investigations on the mechanism underlying the development of HPV-related SPTs are urgently needed.

## Data Availability Statement

The original contributions presented in the study are included in the article, further inquiries can be directed to the corresponding author.

## Ethics Statement

The studies involving human participants were reviewed and approved by Ethics Committee Canton St. Gallen. The patients/participants provided their written informed consent to participate in this.

## Author Contributions

VG contributed to data acquisition, data analysis and interpretation, manuscript writing. LS contributed to data acquisition, data analysis and manuscript editing. MB contributed to study conception and design, data interpretation and overall review. KI, NR, and DH contributed to data acquisition and analysis. SS and MP contributed to study conception and design. WJ contributed to acquisition of data. All authors contributed to the article and approved the submitted version.

## Funding

This research did not receive any specific grant from funding agencies in the public, commercial, or not-for-profit sectors.

## Conflict of Interest

The authors declare that the research was conducted in the absence of any commercial or financial relationships that could be construed as a potential conflict of interest.

## Publisher’s Note

All claims expressed in this article are solely those of the authors and do not necessarily represent those of their affiliated organizations, or those of the publisher, the editors and the reviewers. Any product that may be evaluated in this article, or claim that may be made by its manufacturer, is not guaranteed or endorsed by the publisher.
